# Transceiver Optimization for mmWave Line-of-Sight MIMO Systems Using Hybrid Arrays

**DOI:** 10.3390/mi14020236

**Published:** 2023-01-17

**Authors:** Junwen Deng, Hang Li, Jian Andrew Zhang, Xiaojing Huang, Zhiqun Cheng

**Affiliations:** 1 School of Electronics and Information, Hangzhou Dianzi University, Hangzhou 310018, China; 2 The Global Big Data Technologies Centre, University of Technology Sydney, Sydney 2007, Australia

**Keywords:** line-of-sight, multiple input multiple output, transceiver optimization, hybrid arrays, quantization

## Abstract

The performance of millimeter wave (mmWave) line-of-sight multiple input multiple output (LOS MIMO) systems using hybrid arrays of planar subarrays was studied. We characterized the achievable maximum spatial multiplexing gain for such LOS MIMO systems by the measures of spectral efficiency and effective degree of freedom (EDoF). By proposing a joint plane-wave and spherical-wave-based general 3D channel model, we derived the optimal design parameters in the analog domain, i.e., the optimal subarray separation products, and analyzed their sensitivity on the system performance. We also gave analytical eigenvalue expressions of the equivalent LOS MIMO channel matrix, which are applicable to the case of a non-optimal design, as well as the upper and lower bounds of the EDoF for system performance evaluation. A piecewise uniform quantization codebook was further designed for quantizing phase shifter values in practical applications. The numerical and simulation results show that planar subarrays are superior to traditional arrays in terms of spectral efficiency and EDoF in Ricean fading channels because they are more robust to the change in the communication distance and the deviation from the optimal design. The use of hybrid arrays of planar subarrays effectively removes the limitation of mmWave LOS MIMO systems using traditional arrays, through which, the conventional Rayleigh distance criterion has to be satisfied to achieve the optimal performance.

## 1. Introduction

With the ever increasing demand for data traffic, network throughput and wireless devices, traditional microwave frequency bands have been unable to satisfy the requirements of a fast-growing system capacity and spectral efficiency [1]. Therefore, the millimeter wave (mmWave) with a higher frequency band has recently attracted more attention [2,3,4]. The mmWave communications generally operate at 30 GHz–300 GHz with a wavelength of 1 mm–10 mm, which leads to a relatively small physical size of the antennas. This enables the large-scale multiple input multiple output (MIMO) with hundreds of antennas that can meet the high throughput and coverage needs of future networks to be integrated in a limited space for practical applications [5], and also to compensate for the high path loss of mmWave channels for long-distance communications [6,7].

Unlike the traditional MIMO channels in dense scattering environments, mmWave channels are usually characterized as sparse scattering, and thus go against conventional spatial multiplexing [8,9]. Since the pure line-of-sight (LOS) component generally dominates the mmWave channels, it is of great significance to study LOS MIMO spatial multiplexing by employing well-designed array deployment [10]. Hybrid antenna arrays have proven to be a critical technology of cost-effectively achieving high-capacity and long-range communications [11,12,13]. They will not only enable LOS MIMO without the need for adjusting the spacing of subarrays, but also significantly increase the transmission power and decrease the propagation loss by beamforming to reach longer communication distances [14]. The related works are not yet to be widely developed in LOS MIMO systems using hybrid arrays of analog planar subarrays.

In LOS channels, the reflective paths do not exist or their power can be ignored, which results in a higher probability of an insufficient rank in the MIMO channel matrix. However, a well-designed antenna array can still be used to obtain a high-rank channel matrix and higher spatial multiplexing gain in a pure LOS environment [15]. References [16,17] enhance the spatial multiplexing gain of LOS MIMO systems by adjusting the array diameter in a uniform circular array. The technique based on the optimization of antenna placement in uniform linear digital arrays with respect to mutual information was investigated in [18,19], and then extended to uniform planar arrays in [20]. The optimal antenna separation product was derived for a general 3D geometrical LOS MIMO channel model. It was found that the optimal performance can be obtained only when the system parameters satisfy the Rayleigh distance criterion. In general, this refers to the condition that the communication distance *R* should be equal to or less than the so-called Rayleigh distance, i.e., $R\leq Vd_{t}d_{r}\cos\theta_{t}\cos\theta_{r}/\lambda$, where $d_{t}d_{r}$ is the antenna separation product, λ the carrier wavelength, *V* the maximum number of antennas between the transceiver and $\theta_{t}$ ($\theta_{r}$) the angles of the local spherical coordinate system at the transmitter (receiver).

Although LOS MIMO has been considered to be able to achieve spatial multiplexing, the conventional full digital array LOS MIMO technology is infeasible for use in aerial platforms as the antenna spacing would have to be adjusted according to the communication distance, which is ever-changing. The inflexible adjustment is likely to cause a serious performance loss of spatial multiplexing when the Rayleigh distance criterion cannot be satisfied. To reduce the optimal antenna spacing for achieving full spatial multiplexing, the investigation in [21] added a dielectric medium to the signal transmission path for potentially improving channel conditioning with additional phase shifting. Although the limitation of the Rayleigh distance criterion can be relieved to a certain extent, each change in the transmission distance has to be accompanied by the replacement of dielectric mediums with different shapes and types, which is inflexible and even infeasible in practical applications. The work in [22] introduced an asymmetric linear subarray structure instead of the traditional digital arrays only at the transmitter, which can provide both array gain and spatial multiplexing gain and achieve a trade-off between them. In [23], the design of multiple planar arrays at the transceiver was investigated in LOS MIMO systems, and an algorithm for optimizing the antenna position was proposed to reduce the computational complexity. However, only the spherical wave model was used for the LOS channel component, and the phase shifters matrix was not considered for the beamforming design.

Considering the power consumption and system complexity, radio frequency (RF) analog beamformer/combiners are typically implemented using phase shifters. The phase shifters are controlled digitally, and a finite number of values is usually selected subject to the quantization bits. Various quantization codebook designs or phase shifter structures have been investigated in mmWave MIMO systems [24,25,26]. The study in [24] considered two kinds of RF beamforming codebooks. One is the general quantized beamforming codebook, which is usually designed for rich channels with a uniform quantization on the space of beamforming vectors. The other is the beamsteering codebooks, where the beamforming vectors as spatially matched filters can be parameterized by a simple angle. Its simulation results showed the effect of the number of quantization bits on the spectral efficiency in limited scattering mmWave channels. A two-phase-shifter structure for each coefficient of the RF analog precoder was proposed and analyzed in [25], through which, any precoding coefficients can be represented with a very small quantization error when the number of quantization bits is sufficiently large. To reduce the large quantization error introduced by separated quantization, a joint quantization method that uses a combined codebook of the two phase shifters was proposed in [26]. Although the two-phase-shifter structure can control the beamforming weight flexibly to achieve full multiplexing gain, the doubled number of phase shifters will increase the complexity of hardware implementation. A codebook-based beamforming training method was used for RF precoding weights of subarrays in [22], whereas the impact of codebooks and quantization bits on the system performance was not given.

In this paper, we studied the performance of mmWave LOS MIMO systems employing hybrid arrays of planar subarrays in terms of the spectral efficiency and effective degree of freedom (EDoF), where the EDoF represents the achievable maximum system spatial multiplexing gain. We propose a joint plane-wave and spherical-wave-based general 3D channel model for the LOS MIMO channel, which allows for a non-parallel orientation of hybrid arrays to be employed at the transceiver. The performance in terms of the spectral efficiency and EDoF using hybrid arrays of planar subarrays was analyzed and compared with that of the traditional digital arrays for different deviation factors and Ricean fading channels. The numerical and simulation results show that using the proposed planar subarray structure can still achieve an almost optimal performance even though the parameter design deviates from the Rayleigh distance criterion in a relatively wide range. The main contributions can be summarized as follows.

1. Based on the proposed 3D channel model, the optimal subarray separation products in the vertical and horizontal directions were derived for maximizing the spectral efficiency and EDoF, respectively, and an analysis of the sensitivity to the non-optimal design was performed by means of deviation factors.
2. The theoretical expressions for the eigenvalues of an equivalent LOS MIMO channel matrix were derived as a function of deviation factors, in both cases of optimal and non-optimal designs. The upper and lower bounds of the EDoF are also given.
3. The piecewise uniform quantization codebooks for the phase angles in the beamformer and combiner were designed for LOS MIMO systems, which enables the results to be applied in practical systems with quantized RF phase shifters. Analytical expressions for spectral efficiency using the designed codebooks were also derived. The numerical results demonstrate that the designed codebooks outperform the beamsteering codebook in [24] using the same number of quantization bits in terms of spectral efficiency.

The remainder of this paper is organized as follows. Section 2 introduces the received signal models using hybrid arrays with planar subarrays and performance measures, followed by a joint plane-wave and spherical-wave-based 3D channel model. Section 3 derives the eigenvalue expressions of the equivalent channel matrix considering deviation factors caused by a non-optimal design, and gives the upper and lower bounds of EDoF. Section 4 designs uniform quantization codebooks for phase shifter values employed in practical applications. In Section 5, numerical and simulation results are given to verify our analysis and demonstrate the effects of design parameters on the system performance, before concluding the paper in Section 6.

The following notations are used throughout this paper. $A^{H}$ denotes the conjugate transpose of matrix A; ${∥A∥}_{F}$ is the Frobenius norm of matrix A; $1_{U}$ is the U×U all-ones matrix; $I_{U}$ is the U×U identity matrix; diag(a) denotes a diagonal matrix whose diagonal elements are formed by vector **a**; ${∥a∥}_{2}$ is the 2-norm of vector a, and $a^{T}$ is its transpose; $E\left\{\cdot \right\}$ is used to denote expectation. Further, the notations $\det\left\{\cdot \right\}$, $\mathrm{tr}\left\{\cdot \right\}$ and $\mathrm{rank}\left\{\cdot \right\}$ represent the determinant, trace and rank of $\left\{\cdot \right\}$, respectively.

## 2. System Model

### 2.1. Signal Model

**Figure 1:**
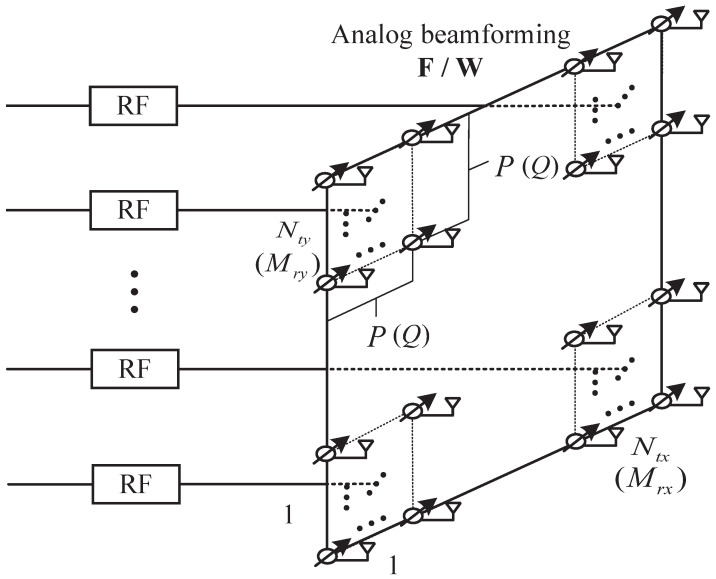
The hybrid arrays with planar subarrays.

Consider a point-to-point LOS MIMO communication system where the hybrid arrays with planar subarrays are used in the transceiver, respectively. As shown in Figure 1, each subarray is connected by an RF chain with multiple phase shifters, which are used to implement analog beamforming. Let the number of subarrays at the transmitter (receiver) be $N=N_{ty}\times N_{tx}$ ($M=M_{ry}\times M_{rx}$), where $N_{ty}$ and $N_{tx}$ ($M_{ry}$ and $M_{rx}$) represent the numbers of vertical and horizontal subarrays, respectively. We assume that each subarray at the transmitter (receiver) contains P×P (Q×Q) antenna elements. Denote the transmitted signal vector as $s\in C^{N\times 1}$ and the normalized complex channel matrix as $H\in C^{MQ^{2}\times NP^{2}}$. Let $F\in C^{NP^{2}\times N}$ and $W\in C^{MQ^{2}\times M}$ denote the phase shifter matrices at the transceiver, respectively. Assuming slowly varying and frequency-flat fading channels (although we only study the narrowband channels in this paper, the proposed approach can be extended to wideband channels by dividing the wideband into multiple narrowbands, e.g., in OFDM systems. The optimal design and analysis in this paper can be applied to each narrowband subcarrier accordingly), the received signal vector $y\in C^{M\times 1}$ after analog beamforming combining for each subarray can be modeled as

(1) $$\begin{array}{c} y=\sqrt{\rho }W^{H}HFs+W^{H}n=\sqrt{\rho }\tilde{H}s+W^{H}n, \end{array}$$

where ρ is the power attenuation coefficient of the transmitted signals over a subchannel between a pair of transmit and receive subarrays, and assumed to be a constant for all subchannels [27,28]. $n\in C^{MQ^{2}\times 1}$ is the additive white Gaussian noise (AWGN) vector with zero mean value and noise power $\sigma_{n}^{2}$. $\tilde{H}\in C^{M\times N}$ denotes the equivalent baseband channel between the transceiver after the LOS MIMO propagation and the analog beamforming.

Assuming that the channel state information is perfectly known at the receiver and equal power transmission is used, the spectral efficiency of the system can be expressed as [29]

(2) $$\begin{array}{c} SE=\;\;\log_{2}\det\left(I_{U}+\frac{\bar{\gamma }}{N}V\right)=\;\;\sum_{k=1}^{U}\log_{2}\left(1+\frac{\bar{\gamma }}{N}\mu_{k}\right)\;\;\;\;\;\mathrm{bits}/s/\mathrm{Hz}, \end{array}$$

where $\bar{\gamma }$ represents the average SNR after analog beamforming combining at the receiver and U=min{M,N}. $\mu_{k}$ is the *k*th eigenvalue of matrix V, and V is expressed as

(3) $$V=\left\{\begin{array}{cc} \tilde{H}{\tilde{H}}^{H}, & M<N\\ {\tilde{H}}^{H}\tilde{H}, & M\geq N. \end{array}\right.$$

The MIMO system can achieve the optimal performance only when its channel is equivalent to multiple independent single input single output (SISO) subchannels. Therefore, we characterize the achievable spatial multiplexing gain of the system by maximizing the EDoF as [23,30]

(4) $$\begin{array}{c} EDoF=\;\;\frac{d\left(SE\left(2^{\delta }\bar{\gamma }\right)\right)}{d\delta }{|}_{\delta =0}=\;\;\sum_{k=1}^{U}\frac{\bar{\gamma }\mu_{k}}{N+\bar{\gamma }\mu_{k}}. \end{array}$$

From (2) and (4), we can see that the spectral efficiency and EDoF are not only determined by the eigenvalue $\mu_{k}$ of matrix V, but also by the number of subarrays at the transmitter and the average SNR at the receiver. When the product of the eigenvalue $\mu_{k}$ and the average SNR is far greater than *N*, a 3 dB increase in SNR leads to an approximate increase in *U* bits/s/Hz in spectral efficiency [30]. The work in [19] revealed that the optimal EDoF and system spectral efficiency can be achieved when any two columns or rows of the channel matrix satisfy orthogonality, i.e., all eigenvalues of the channel matrix are equal. This orthogonality criterion will be used in Section 3.

### 2.2. Channel Model

A Ricean channel matrix can be modeled as the sum of an LOS component and an NLOS component in [31,32]. Assume that the normalized channel matrix H is Ricean and can be expressed as

(5) $$\begin{array}{c} H=\sqrt{\frac{K}{1+K}}H_{LOS}+\sqrt{\frac{1}{1+K}}H_{NLOS}, \end{array}$$

where the Ricean factor *K* represents the ratio between the power of the two components. In this paper, we focused on the pure LOS channel in an MIMO system, which indicates that K=+∞. $H_{LOS}$ is hereinafter referred to as H unless stated otherwise. As shown in [33], when the antenna array is appropriately configured, the pure LOS MIMO channel matrix is a high-rank matrix with a number of nonzero eigenvalues, which leads to a high spectral efficiency.

**Figure 2:**
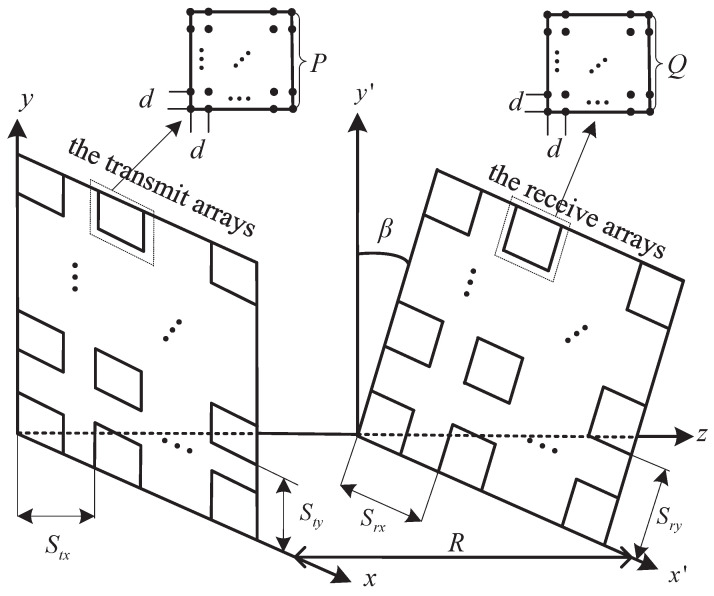
Channel model of signal transmission.

As illustrated in Figure 2, we assume that the separations of adjacent subarrays along the vertical and horizontal directions at the transmitter (receiver) are $S_{tx}$ and $S_{ty}$ ($S_{rx}$ and $S_{ry}$), respectively, and that the spacings of adjacent antenna elements in each subarray along the vertical and horizontal directions are *d* for simplicity. The transmit arrays are placed on the xy plane, β is the angle between the xy plane and the plane of the receive arrays and *R* represents the horizontal communication distance between the bottom of the transceiver arrays. As in [34,35], due to $d\ll (S_{tx},S_{ty},S_{rx},S_{ry})\ll R$, we used the plane-wave model for the received signals within a subarray, but the spherical-wave model for the received signals between different subarrays, which is reflected in the distance between a pair of antenna elements at the transceiver.

Therefore, an element of the channel matrix H, $h_{r,t}$ ($r=0,1,\ldots ,MQ^{2}-1$, $t=0,1,\ldots ,NP^{2}-1$), in the planar subarray structure can be represented as

(6) $$\begin{array}{c} h_{r,t}=e^{j\frac{2\pi }{\lambda }l_{r,t}}, \end{array}$$

where λ is the carrier wavelength, $l_{r,t}$ represents the distance between the *t*th transmitting antenna element and the *r*th receiving antenna element and

$$\begin{array}{c} r=\left(m_{y}M_{rx}+m_{x}\right)Q^{2}+y_{mr}Q+x_{mr}, \end{array}$$

(7) $$\begin{array}{c} t=\left(n_{y}N_{tx}+n_{x}\right)P^{2}+y_{nt}P+x_{nt}, \end{array}$$

where $m_{x}\;\left(m_{y}\right)=0,1,\ldots ,M_{rx}-1\;\left(M_{ry}-1\right)$, $n_{x}\;\left(n_{y}\right)=0,1,\ldots ,N_{tx}-1\;\left(N_{ty}-1\right)$, $x_{mr}\;\left(y_{mr}\right)=0,1,\ldots ,Q-1$ and $x_{nt}\;\left(y_{nt}\right)=0,1,\ldots ,P-1$. Let $m=m_{y}M_{rx}+m_{x}$ and $n=n_{y}N_{tx}+n_{x}$. $l_{r,t}$ can be rewritten as [36]

(8) $$\begin{array}{cc} l_{r,t}= & \;\;l_{m,n}+d\cdot x_{nt}\sin\theta_{nt}\cos\varphi_{nt}+d\cdot y_{nt}\sin\theta_{nt}\sin\varphi_{nt}\\ \; & \;\;+d\cdot x_{mr}\sin\theta_{mr}\cos\varphi_{mr}+d\cdot y_{mr}\sin\theta_{mr}\sin\varphi_{mr}, \end{array}$$

where $l_{m,n}$ represents the distance between a reference element in the *n*th transmit subarray and that in the *m*th receive subarray. In this paper, we took the antenna element located at the origin of the coordinate axis in the first subarray as the reference element. $\theta_{nt}$ and $\varphi_{nt}$ ($\theta_{mr}$ and $\varphi_{mr}$) are the signal elevation and azimuth angles away from the *n*th transmit subarray (to the *m*th receive subarray), respectively. $l_{m,n}$ is further calculated by

(9) $$\begin{array}{cc} l_{m,n}= & \;\;{\left[{\left(m_{x}S_{rx}-n_{x}S_{tx}\right)}^{2}+{\left(R+m_{y}S_{ry}\sin\beta \right)}^{2}+{\left(m_{y}S_{ry}\cos\beta -n_{y}S_{ty}\right)}^{2}\right]}^{\frac{1}{2}}\\ = & \;\;\left(R+m_{y}S_{ry}\sin\beta \right){\left[1\;+\;\frac{{\left(m_{x}S_{rx}\;-\;n_{x}S_{tx}\right)}^{2}\;+\;{\left(m_{y}S_{ry}\cos\beta \;-\;n_{y}S_{ty}\right)}^{2}}{{\left(R+m_{y}S_{ry}\sin\beta \right)}^{2}}\right]}^{\frac{1}{2}}\\ \overset{\left(a\right)}{\approx } & \;\;R+m_{y}S_{ry}\sin\beta +\frac{{\left(m_{x}S_{rx}-n_{x}S_{tx}\right)}^{2}+{\left(m_{y}S_{ry}\cos\beta -n_{y}S_{ty}\right)}^{2}}{2R}, \end{array}$$

where (a) holds due to $R\gg (S_{tx},S_{ty},S_{rx},S_{ry})$ by using the McLaughlin approximation formula; that is, when x→0, ${\left(1+x\right)}^{1/a}=1+\frac{1}{a}x$.

As a result, the channel matrix H can be expressed as

(10) $$\begin{array}{c} H=\left[\begin{array}{ccc} H_{0,0} & \ldots & H_{0,(N-1)}\\ \vdots & \ddots & \vdots \\ H_{(M-1),0} & \ldots & H_{\left(M-1),(N-1\right)} \end{array}\right], \end{array}$$

where the subchannel matrix

$$\begin{array}{c} H_{m,n}=h_{m,n}\left[\begin{array}{ccc} {\hat{h}}_{0,0} & \ldots & {\hat{h}}_{0,(P^{2}-1)}\\ \vdots & \ddots & \vdots \\ {\hat{h}}_{(Q^{2}-1),0} & \ldots & {\hat{h}}_{\left(Q^{2}-1\right),\left(P^{2}-1\right)} \end{array}\right] \end{array}$$

and

$$\begin{array}{cc} {\hat{h}}_{q_{m},p_{n}}= & \;\;\exp(jd^{\prime }[q_{m}^{x}\sin\theta_{mr}\cos\varphi_{mr}+q_{m}^{y}\sin\theta_{mr}\sin\varphi_{mr}\\ \; & \;\;+p_{n}^{x}\sin\theta_{nt}\cos\varphi_{nt}+p_{n}^{y}\sin\theta_{nt}\sin\varphi_{nt}]), \end{array}$$

where $q_{m}=q_{m}^{y}Q+q_{m}^{x}$, $p_{n}=p_{n}^{y}P+p_{n}^{x}$, $q_{m}^{x}\;\left(q_{m}^{y}\right)=0,1,\ldots Q-1$, $p_{n}^{x}\;\left(p_{n}^{y}\right)=0,1,\ldots P-1$ and $d^{\prime }=\frac{2\pi }{\lambda }d$.

As the equivalent channel matrix $\tilde{H}$ includes the analog beamforming matrix F and combining matrix W, we need to consider their influence on the optimal design in the analog domain of LOS MIMO systems with planar subarrays. F and W can be expressed as

(11) $$\begin{array}{c} F=\mathrm{diag}\{f_{0},f_{1},\ldots ,f_{N_{tx}-1},f_{N_{tx}},\ldots ,f_{N-1}\} \end{array}$$

and

(12) $$\begin{array}{c} W=\mathrm{diag}\{w_{0},w_{1},\ldots ,w_{M_{rx}-1},w_{M_{rx}},\ldots ,w_{M-1}\}, \end{array}$$

respectively. In this paper we used a single beam for each subarray, and therefore the normalized weight vectors $f_{n}$ and $w_{m}$ of the *n*th transmit subarray and the *m*th receive subarray are written as

(13) $$\begin{array}{cc} f_{n}=\;\; & \frac{1}{P}[1,\ldots ,\exp\left(-jd^{\prime }\left(p_{n}^{x}\sin\alpha_{nt}\cos\phi_{nt}+p_{n}^{y}\sin\alpha_{nt}\sin\phi_{nt}\right)\right),\\ \; & \;\;\;\;\;\;\;\;\ldots ,\exp\left(-j\left(P-1\right)d^{\prime }\left(\sin\alpha_{nt}\cos\phi_{nt}+\sin\alpha_{nt}\sin\phi_{nt}\right)\right){]}^{T} \end{array}$$

and

(14) $$\begin{array}{cc} w_{m}=\;\; & \frac{1}{Q}[1,\ldots ,\exp\left(jd^{\prime }\left(q_{m}^{x}\sin\alpha_{mr}\cos\phi_{mr}+q_{m}^{y}\sin\alpha_{mr}\sin\phi_{mr}\right)\right),\\ \; & \;\;\;\;\;\;\;\;\ldots ,\exp\left(j\left(Q\;-\;1\right)d^{\prime }\left(\sin\alpha_{mr}\cos\phi_{mr}\;+\;\sin\alpha_{mr}\sin\phi_{mr}\right)\right){]}^{T}, \end{array}$$

which allow the main beam of the *n*th transmit subarray (the *m*th receive subarray) to be directed towards the direction represented by the angles ($\alpha_{nt},\phi_{nt}$) (($\alpha_{mr},\phi_{mr}$)).

## 3. Optimal Design of Planar Subarrays

### 3.1. Analysis of Eigenvalues

As stated in [19], for a conventional LOS MIMO system with linear digital arrays, the optimal product of the inter-antenna distances at the transceiver can be acquired when different columns or rows of the channel matrix satisfy orthogonality, which is used to maximize the spectral efficiency and EDoF. Inspired by this, we investigated the effect of the planar subarray separations at the transceiver on the system spatial multiplexing performance.

We first considered the situation where *M* is larger than *N*. Although the rank of the equivalent channel matrix $\tilde{H}$ is *U* in general, only the power allocated to EDoF out of *U* can be devoted to the spectral efficiency. The correlation among the components of $\tilde{H}$ becomes increasingly high with the reduced adjacent subarray separations at the transceiver, which can cause a loss of system spectral efficiency and EDoF. In order to reduce the correlation between RF chains, we aimed to design the beamforming matrices such that different columns of $\tilde{H}$ are orthogonal, i.e., ${\tilde{H}}_{n_{1}}^{H}{\tilde{H}}_{n_{2}}=0$ ($n_{1}\neq n_{2}$).

From (10), (13) and (14), the orthogonality requires that

(15) $$\begin{array}{cc} \; & {\tilde{H}}_{n_{1}}^{H}{\tilde{H}}_{n_{2}}\\ = & \sum_{m=0}^{M-1}f_{n_{1}}^{H}H_{m,n_{1}}^{H}w_{m}w_{m}^{H}H_{m,n_{2}}f_{n_{2}}\\ = & \sum_{m=0}^{M-1}\frac{1}{P^{2}Q^{2}}h_{m,n_{1}}^{H}h_{m,n_{2}}\sum_{q_{m}=0}^{Q^{2}-1}\exp[-jd^{\prime }\left(A_{m}q_{m}^{x}+B_{m}q_{m}^{y}\right)]\\ \;\;\;\;\;\; & \cdot \sum_{p_{n_{1}}=0}^{P^{2}-1}\exp[-jd^{\prime }\left(C_{n_{1}}p_{n_{1}}^{x}+D_{n_{1}}p_{n_{1}}^{y}\right)]\sum_{q_{m}=0}^{Q^{2}-1}\exp[jd^{\prime }\left(A_{m}q_{m}^{x}+B_{m}q_{m}^{y}\right)]\\ \;\;\;\;\;\; & \cdot \sum_{p_{n_{2}}=0}^{P^{2}-1}\exp[jd^{\prime }\left(C_{n_{2}}p_{n_{2}}^{x}+D_{n_{2}}p_{n_{2}}^{y}\right)]\\ = & 0, \end{array}$$

where

$$\begin{array}{c} A_{m}=\sin\theta_{mr}\cos\varphi_{mr}-\sin\alpha_{mr}\cos\phi_{mr}, \end{array}$$

$$\begin{array}{c} B_{m}=\sin\theta_{mr}\sin\varphi_{mr}-\sin\alpha_{mr}\sin\phi_{mr}, \end{array}$$

$$\begin{array}{c} C_{n_{k}}=\sin\theta_{n_{k}t}\cos\varphi_{n_{k}t}-\sin\alpha_{n_{k}t}\cos\phi_{n_{k}t} \end{array}$$

and

$$\begin{array}{c} D_{n_{k}}=\sin\theta_{n_{k}t}\sin\varphi_{n_{k}t}-\sin\alpha_{n_{k}t}\sin\phi_{n_{k}t},\;\;\left(k=1,2\right). \end{array}$$

Since $\sum_{q_{m}=0}^{Q^{2}-1}\exp[-jd^{\prime }\left(A_{m}q_{m}^{x}+B_{m}q_{m}^{y}\right)]$ and the last three summations in (15) are not guaranteed to be zero, we let

(16) $$\begin{array}{c} {\tilde{H}}_{n_{1}}^{H}{\tilde{H}}_{n_{2}}=\sum_{m=0}^{M-1}h_{m,n_{1}}^{H}h_{m,n_{2}}=\sum_{m=0}^{M-1}\exp\left(j\frac{2\pi }{\lambda }\left(l_{m,n_{2}}-l_{m,n_{1}}\right)\right)=0. \end{array}$$

Substituting (9) into (16), we can obtain

(17) $$\begin{array}{cc} \; & \sum_{m=0}^{M-1}h_{m,n_{1}}^{H}h_{m,n_{2}}\\ = & \sum_{m_{y}=0}^{M_{ry}-1}\sum_{m_{x}=0}^{M_{rx}-1}\exp\left(j\frac{2\pi }{\lambda }\frac{\left[m_{x}\left(n_{1_{x}}-n_{2_{x}}\right)S_{tx}S_{rx}\right]}{R}\right)\exp\left(j\frac{2\pi }{\lambda }\frac{\left[m_{y}\left(n_{1_{y}}-n_{2_{y}}\right)S_{ty}S_{ry}\cos\beta \right]}{R}\right)\\ = & \frac{\sin\left(\frac{M_{ry}\pi }{\lambda R}\left(n_{1_{y}}-n_{2_{y}}\right)S_{ty}S_{ry}\cos\beta \right)}{\sin\left(\frac{\pi }{\lambda R}\left(n_{1_{y}}-n_{2_{y}}\right)S_{ty}S_{ry}\cos\beta \right)}\frac{\sin\left(\frac{M_{rx}\pi }{\lambda R}\left(n_{1_{x}}-n_{2_{x}}\right)S_{tx}S_{rx}\right)}{\sin\left(\frac{\pi }{\lambda R}\left(n_{1_{x}}-n_{2_{x}}\right)S_{tx}S_{rx}\right)}\overset{\left(b\right)}{=}0. \end{array}$$

There are infinite solutions for (b) in (17), but we only chose the smallest subarray separation products in the vertical and horizontal directions as they are of most interest in practical systems. Therefore, the optimal subarray separation products are derived by

(18) $$\begin{array}{c} S_{ry}S_{ty}=\frac{\lambda R}{M_{ry}\cos\beta },\;\;\;\;\;\;\;\;\;\;\;\;\;\;\;\;S_{rx}S_{tx}=\frac{\lambda R}{M_{rx}}, \end{array}$$

which enable the maximal spatial multiplexing gain of the system with planar subarrays. A similar result can be obtained by substituting $N_{ty}$ and $N_{tx}$ for $M_{ry}$ and $M_{rx}$ in (18) when *M* is smaller than *N*. In general, the optimal subarray separation products for planar subarrays can be given by

(19) $$\begin{array}{c} S_{ry}S_{ty}=\frac{\lambda R}{V_{1}\cos\beta },\;\;\;\;\;\;\;\;\;\;\;\;\;\;\;\;S_{rx}S_{tx}=\frac{\lambda R}{V_{2}}, \end{array}$$

where

$$\begin{array}{c} \left(V_{1},V_{2}\right)=\left\{\begin{array}{c} (M_{ry},M_{rx}),\;\;\;\;\;\;\;\;\;\;\;\;M\geq N\\ (N_{ty},N_{tx}),\;\;\;\;\;\;\;\;\;\;\;\;\;\;\;M<N. \end{array}\right. \end{array}$$

### 3.2. Analysis of Sensitivity to Displacement

In order to investigate the sensitivity of LOS MIMO systems with planar subarrays to the deviations from the optimal planar subarray design in (19), we introduced the deviation factors, defined as the ratio of the optimal subarray separation product to the actual subarray separation product,

(20) $$\begin{array}{c} \eta_{1}=\frac{\lambda R}{S_{ry}S_{ty}\cos\beta V_{1}},\;\;\;\;\;\;\;\;\;\;\;\;\eta_{2}=\frac{\lambda R}{S_{rx}S_{tx}V_{2}}, \end{array}$$

which indicate that the actual vertical (horizontal) subarray separation product is smaller than the optimal one if $\eta_{1}$ ($\eta_{2}$) is larger than one.

From (2) and (4), we can see that the system spectral efficiency and EDoF depend on the eigenvalues of V. For ease of illustration, we considered the case of $N_{ty}=N_{tx}=M_{ry}=M_{rx}=P=Q=2$, and thus the equivalent transmission channel matrix $\tilde{H}$ can be expressed as

(21) $$\begin{array}{c} \tilde{H}=W^{H}HF=\left[\begin{array}{cccc} {\tilde{H}}_{0,0} & {\tilde{H}}_{0,1} & {\tilde{H}}_{0,2} & {\tilde{H}}_{0,3}\\ {\tilde{H}}_{1,0} & {\tilde{H}}_{1,1} & {\tilde{H}}_{1,2} & {\tilde{H}}_{1,3}\\ {\tilde{H}}_{2,0} & {\tilde{H}}_{2,1} & {\tilde{H}}_{2,2} & {\tilde{H}}_{2,3}\\ {\tilde{H}}_{3,0} & {\tilde{H}}_{3,1} & {\tilde{H}}_{3,2} & {\tilde{H}}_{3,3} \end{array}\right], \end{array}$$

where

$$\begin{array}{c} {\tilde{H}}_{m,n}=w_{m}^{H}H_{m,n}f_{n}. \end{array}$$

When the main beam direction of the *n*th transmit subarray (the *m*th receive subarray) satisfies ($\alpha_{nt}$,$\phi_{nt}$) = ($\theta_{nt}$,$\varphi_{nt}$) (($\alpha_{mr}$,$\phi_{mr}$) = ($\theta_{mr}$,$\varphi_{mr}$)), substituting (21) into (3) leads to

(22) $$\begin{array}{c} V=P^{2}Q^{2}\cdot \left[\begin{array}{cccc} G_{0,0} & G_{0,1} & G_{0,2} & G_{0,3}\\ G_{1,0} & G_{1,1} & G_{1,2} & G_{1,3}\\ G_{2,0} & G_{2,1} & G_{2,2} & G_{2,3}\\ G_{3,0} & G_{3,1} & G_{3,2} & G_{3,3} \end{array}\right], \end{array}$$

where

$$\begin{array}{c} G_{n_{1},n_{2}}=\sum_{m=0}^{M-1}h_{m,n_{1}}^{H}h_{m,n_{2}}=\sum_{m=0}^{M-1}\exp\left(j\frac{2\pi }{\lambda }\left(l_{m,n_{2}}-l_{m,n_{1}}\right)\right). \end{array}$$

Substituting (9) into (22), we can rewrite V as

$$V=P^{2}Q^{2}\left[\begin{array}{cccc} V_{1}V_{2} & \;\;\;\;V_{2}X_{1}Y_{2}Z_{2} & \;\;\;\;V_{1}X_{2}Y_{1}Z_{1} & \;\;\;\;X_{1}X_{2}Y_{1}Y_{2}Z_{1}Z_{2}\\ V_{2}X_{1}^{-1}Y_{2}^{-1}Z_{2} & \;\;\;\;V_{1}V_{2} & \;\;\;\;X_{1}^{-1}X_{2}Y_{1}Y_{2}^{-1}Z_{1}Z_{2} & \;\;\;\;V_{1}X_{2}Y_{1}Z_{1}\\ V_{1}X_{2}^{-1}Y_{1}^{-1}Z_{1} & \;\;\;\;X_{1}X_{2}^{-1}Y_{1}^{-1}Y_{2}Z_{1}Z_{2} & \;\;\;\;V_{1}V_{2} & \;\;\;\;V_{2}X_{1}Y_{2}Z_{2}\\ X_{1}^{-1}X_{2}^{-1}Y_{1}^{-1}Y_{2}^{-1}Z_{1}Z_{2} & \;\;\;\;V_{1}X_{2}^{-1}Y_{1}^{-1}Z_{1} & \;\;\;\;V_{2}X_{1}^{-1}Y_{2}^{-1}Z_{2} & \;\;\;\;V_{1}V_{2} \end{array}\right],$$

where

$$\begin{array}{c} X_{1}=\exp\left(j\frac{\pi }{\lambda R}\cdot S_{tx}^{2}\right),\;\;\;\;\;\;\;\;X_{2}=\exp\left(j\frac{\pi }{\lambda R}\cdot S_{ty}^{2}\right) \end{array}$$

and

$$\begin{array}{c} Y_{i}=\exp\left(-j\frac{\pi }{2\eta_{i}}\right),\;\;\;\;\;\;\;\;Z_{i}=\frac{\sin(\pi /\eta_{i})}{\sin(\pi /\left(2\eta_{i}\right))}\;\;\;\;\;\left(i=1,2\right). \end{array}$$

We find that the eigenvalues of V are equivalent to the eigenvalues of $V^{\prime }$, which is given by

$$\begin{array}{c} V^{\prime }=P^{2}Q^{2}\cdot \left[\begin{array}{cccc} V_{1}V_{2} & V_{2}Z_{2} & V_{1}Z_{1} & Z_{1}Z_{2}\\ V_{2}Z_{2} & V_{1}V_{2} & Z_{1}Z_{2} & V_{1}Z_{1}\\ V_{1}Z_{1} & Z_{1}Z_{2} & V_{1}V_{2} & V_{2}Z_{2}\\ Z_{1}Z_{2} & V_{1}Z_{1} & V_{2}Z_{2} & V_{1}V_{2} \end{array}\right]. \end{array}$$

Then, the eigenvalues can be found as

(23) $$\begin{array}{c} \mu_{1}=V_{1}V_{2}P^{2}Q^{2}\;-\;P^{2}Q^{2}\left(V_{1}Z_{1}+V_{2}Z_{2}\right)\;+\;P^{2}Q^{2}Z_{1}Z_{2}, \end{array}$$

(24) $$\begin{array}{c} \mu_{2}=V_{1}V_{2}P^{2}Q^{2}\;+\;P^{2}Q^{2}\left(V_{1}Z_{1}-V_{2}Z_{2}\right)\;-\;P^{2}Q^{2}Z_{1}Z_{2}, \end{array}$$

(25) $$\begin{array}{c} \mu_{3}=V_{1}V_{2}P^{2}Q^{2}\;+\;P^{2}Q^{2}\left(V_{1}Z_{1}+V_{2}Z_{2}\right)\;+\;P^{2}Q^{2}Z_{1}Z_{2} \end{array}$$

and

(26) $$\begin{array}{c} \mu_{4}=V_{1}V_{2}P^{2}Q^{2}\;-\;P^{2}Q^{2}\left(V_{1}Z_{1}-V_{2}Z_{2}\right)\;-\;P^{2}Q^{2}Z_{1}Z_{2}. \end{array}$$

### 3.3. Upper and Lower Bounds of the EDoF Estimation

It is shown in (4) that the EDoF is determined by the distribution of eigenvalues of V. The sum of the eigenvalues can be calculated as

$$\begin{array}{c} \begin{array}{c} \sum_{i=1}^{U}\mu_{i}=\mathrm{tr}\left({\tilde{H}}^{H}\tilde{H}\right)=\sum_{m=0}^{M-1}\sum_{n=0}^{N-1}{∥w_{m}^{H}H_{m,n}f_{n}∥}_{F}^{2}\\ \;\;\;\;\;\;\;\;\;\;\;\;\;\;\;\;\leq \sum_{m=0}^{M-1}\sum_{n=0}^{N-1}{∥w_{m}^{H}∥}_{2}^{2}{∥H_{m,n}∥}_{F}^{2}{∥f_{n}∥}_{2}^{2}=NMP^{2}Q^{2}, \end{array} \end{array}$$

where the equal sign holds only when the vector $w_{m}^{H}$ ($f_{n}$) is linearly dependent on each column (row) of the subchannel $H_{m,n}$, which corresponds to the case that all eigenvalues in (23)–(26) are equal to $V_{1}V_{2}P^{2}Q^{2}$ when $\eta_{1}=\eta_{2}=0$ dB.

When the subarray separations at the transceiver satisfy (19), i.e., ${\tilde{H}}^{H}\tilde{H}=MP^{2}Q^{2}I_{N}$, different columns or rows of the equivalent channel matrix meet the orthogonality condition, which is equivalent to *U* independent SISO subchannels. In this case, rank(V)=U and all eigenvalues of V are equal to $NMP^{2}Q^{2}/U$, which corresponds to the maximum EDoF given by

(27) $$\begin{array}{c} EDoF_{\max}=\sum_{k=1}^{U}\frac{\bar{\gamma }\mu_{k}}{N+\bar{\gamma }\mu_{k}}=\frac{\bar{\gamma }MP^{2}Q^{2}}{1+\bar{\gamma }\frac{MP^{2}Q^{2}}{U}}. \end{array}$$

When the separations between adjacent subarrays are zero, i.e., ${\tilde{H}}^{H}\tilde{H}=MP^{2}Q^{2}1_{N}$, the RF equivalent channel is completely correlated, and becomes equivalent to a SISO channel. In this case, rank(V)=1, and there is a unique eigenvalue $\mu_{1}=NMP^{2}Q^{2}$, which corresponds to the minimum EDoF given by

(28) $$\begin{array}{c} EDoF_{\min}=\frac{\bar{\gamma }\mu_{1}}{N+\bar{\gamma }\mu_{1}}=\frac{\bar{\gamma }MP^{2}Q^{2}}{1+\bar{\gamma }MP^{2}Q^{2}}. \end{array}$$

## 4. Quantization Codebook Design for Phase Shifters

The continuous and complete phase angle information is usually assumed to be available at the transceiver for RF phase shifter configuration. However, the phase shifters for the analog beamformer/combiner are implemented by discrete phase shifts due to the constraints on the RF hardware in a practical system, and thus the analog beamforming/combining vectors can only take specific values selected from the given quantization codebooks, as they can be easily implemented at a low cost in practical applications, though the spectral efficiency may be lower than that without quantization.

Most of the existing quantization codebooks are designed for traditional MIMO with rich scattering channels, and yet they are not applicable to LOS MIMO channels with few scatterers. According to the introduced channel model, we designed beamsteering codebooks $F_{t}$ ($F_{r}$) to uniformly quantize the phase angle $\alpha_{t}$ ($\alpha_{r}$) of the beamformer (combiner) using $N_{b}$ ($M_{b}$) bits for LOS MIMO channels, which are given by

(29) $$\begin{array}{c} F_{t}:\left\{{\alpha_{t}}_{\min}+\frac{\Delta \alpha_{t}}{2^{N_{b}+1}},{\alpha_{t}}_{\min}+\frac{3\Delta \alpha_{t}}{2^{N_{b}+1}},\ldots ,{\alpha_{t}}_{\max}-\frac{\Delta \alpha_{t}}{2^{N_{b}+1}}\right\} \end{array}$$

and

(30) $$\begin{array}{c} F_{r}:\left\{{\alpha_{r}}_{\min}+\frac{\Delta \alpha_{r}}{2^{M_{b}+1}},{\alpha_{r}}_{\min}+\frac{3\Delta \alpha_{r}}{2^{M_{b}+1}},\ldots ,{\alpha_{r}}_{\max}-\frac{\Delta \alpha_{r}}{2^{M_{b}+1}}\right\}, \end{array}$$

respectively, where ${\alpha_{t}}_{\min}$ and ${\alpha_{t}}_{\max}$ (${\alpha_{r}}_{\min}$ and ${\alpha_{r}}_{\max}$) represent the minimum and maximum achievable phase angles in the beamformer (combiner) before quantization, respectively; $\Delta \alpha_{t}={\alpha_{t}}_{\max}-{\alpha_{t}}_{\min}$ and $\Delta \alpha_{r}={\alpha_{r}}_{\max}-{\alpha_{r}}_{\min}$. The quantization codebooks $W_{t}$ and $W_{r}$ of phase angles $\phi_{t}$ and $\phi_{r}$ have the same expression forms.

In the following, we characterize the effect of quantization bits on the spectral efficiency. Without a loss of generality, we assume that the elevation and azimuth angles of transmitted and received signals are evenly distributed in $\left[0,\pi \right]$ and $N_{b}=M_{b}=b$, and thus the average spectral efficiency after quantization can be expressed as

(31) $$\begin{array}{c} SE\left(b\right)=\;\;E\left\{f\left(\theta_{t},\theta_{r},\varphi_{t},\varphi_{r};N\right)\right\}=\;\;\frac{1}{\pi^{4}}\int_{0}^{\pi }\;\;\;\int_{0}^{\pi }\;\;\;\int_{0}^{\pi }\;\;\;\int_{0}^{\pi }\;\;\;f\left(\theta_{t},\theta_{r},\varphi_{t},\varphi_{r};N\right)d\theta_{t}d\theta_{r}d\varphi_{t}d\varphi_{r}, \end{array}$$

where $f(\theta_{t},\theta_{r},\varphi_{t},\varphi_{r};N)$ denotes the system spectral efficiency given ($\theta_{t},\theta_{r},\varphi_{t},\varphi_{r}$) when the four designed codebooks $N=\{F_{t},F_{r},W_{t},W_{r}\}$ are used. Note that the quantized phase angles in the codebooks are selected such that an estimated phase angle has the closest distance among the quantized ones in their respective codebook.

For ease of illustration, we assume that the phase shifters for phase angles $\phi_{t}$ and $\phi_{r}$ are implemented by continuous phase shifts, i.e., $\phi_{t}=\varphi_{t}$ and $\phi_{r}=\varphi_{r}$. The minimum and maximum achievable phase angles are assumed to be 0 and π for $\alpha_{t}$ and $\alpha_{r}$, respectively, and the corresponding codebook is $\left[\frac{\pi }{2^{b+1}},\frac{3\pi }{2^{b+1}},\ldots ,\pi -\frac{\pi }{2^{b+1}}\right]$ from (29) and (30). Therefore, for a 2×2 subarray’s pure LOS channel with $\eta_{1}=\eta_{2}=0$ dB, (31) becomes

(32) $$\begin{array}{cc} SE(b)= & \;\;\frac{1}{\pi^{2}}\int_{0}^{\pi }\;\;\;\int_{0}^{\pi }f(\theta_{t},\theta_{r};F_{t},F_{r})d\theta_{t}d\theta_{r}\\ = & \;\;\frac{U}{\pi^{2}}\int_{0}^{\pi }\;\;\int_{0}^{\pi }\log_{2}\left(1+\frac{\bar{\gamma }}{N}\cdot \mu \left(\theta_{t},\theta_{r};F_{t},F_{r}\right)\right)d\theta_{t}d\theta_{r}\\ \overset{\left(a\right)}{\approx } & \;\;\frac{U}{\pi^{2}}\;\;\sum_{c_{1}=0}^{2^{b}-1}\;\sum_{c_{2}=0}^{2^{b}-1}\;\int_{\frac{c_{1}\pi }{2^{b}}}^{\frac{(c_{1}+1)\pi }{2^{b}}}\int_{\frac{c_{2}\pi }{2^{b}}}^{\frac{(c_{2}+1)\pi }{2^{b}}}\left[\frac{k_{b}\bar{\gamma }}{N}f_{1}\;\left(\;\theta_{t};\;\frac{(2c_{1}\;+\;1)\pi }{2^{b+1}}\;\right)\;\right.\\ \; & \left.\cdot f_{2}\left(\theta_{r};\frac{(2c_{2}+1)\pi }{2^{b+1}}\right)+a_{b}\right]d\theta_{t}d\theta_{r}\\ = & \;\;U\cdot a_{b}+\frac{U}{\pi^{2}}\cdot \frac{k_{b}\bar{\gamma }}{N}\sum_{c_{1}=0}^{2^{b}-1}\sum_{c_{2}=0}^{2^{b}-1}\\ \; & \left[F_{1}\;\left(\;\frac{(c_{1}\;+\;1)\pi }{2^{b}};\;\frac{(2c_{1}\;+\;1)\pi }{2^{b+1}}\;\right)\;\;-\;F_{1}\;\left(\;\frac{c_{1}\pi }{2^{b}};\;\frac{(2c_{1}\;+\;1)\pi }{2^{b+1}}\;\right)\;\right]\\ \; & \left[F_{2}\;\left(\;\frac{(c_{2}\;+\;1)\pi }{2^{b}};\;\frac{(2c_{2}\;+\;1)\pi }{2^{b+1}}\;\right)\;\;-\;F_{2}\;\left(\;\frac{c_{2}\pi }{2^{b}};\;\frac{(2c_{2}\;+\;1)\pi }{2^{b+1}}\;\right)\;\right], \end{array}$$

where the specific expressions of $f_{1}\left(\theta_{t};\frac{(2c_{1}+1)\pi }{2^{b+1}}\right)$ and $f_{2}\left(\theta_{r};\frac{(2c_{2}+1)\pi }{2^{b+1}}\right)$ are shown as

$$\begin{array}{c} f_{1}\left(\theta_{t};\frac{(2c_{1}+1)\pi }{2^{b+1}}\right)={\left|\sum_{p=0}^{P^{2}-1}\exp\left(jd^{\prime }\left(\sin\theta_{t}-\sin\left(\frac{(2c_{1}+1)\pi }{2^{b+1}}\right)\right)\left(p^{x}\cos\varphi_{t}+p^{y}\sin\varphi_{t}\right)\right)\right|}^{2},\\ f_{2}\left(\theta_{r};\frac{(2c_{2}+1)\pi }{2^{b+1}}\right)={\left|\sum_{q=0}^{Q^{2}-1}\exp\left(jd^{\prime }\left(\sin\theta_{r}-\sin\left(\frac{(2c_{2}+1)\pi }{2^{b+1}}\right)\right)\left(q^{x}\cos\varphi_{r}+q^{y}\sin\varphi_{r}\right)\right)\right|}^{2}. \end{array}$$

Their integral primitive functions are represented as $F_{1}\left(\theta_{t};\frac{(2c_{1}+1)\pi }{2^{b+1}}\right)$ and $F_{2}\left(\theta_{r};\frac{(2c_{2}+1)\pi }{2^{b+1}}\right)$, respectively. In (32), the approximation (a) holds because $\log_{2}\left(1+\frac{\bar{\gamma }}{N}\mu \right)$ can be approximated as linear functions of μ, i.e., $k_{b}\cdot \frac{\bar{\gamma }}{N}\mu +a_{b}$ for different numbers of quantization bits when $\frac{\bar{\gamma }}{N}\cdot \mu \gg 1$. The constants $k_{b}$ and $a_{b}$ can be calculated offline given other parameters. For a given set of parameters, the theoretical results of (32) can be obtained by high-precision numerical integration.

**Figure 3:**
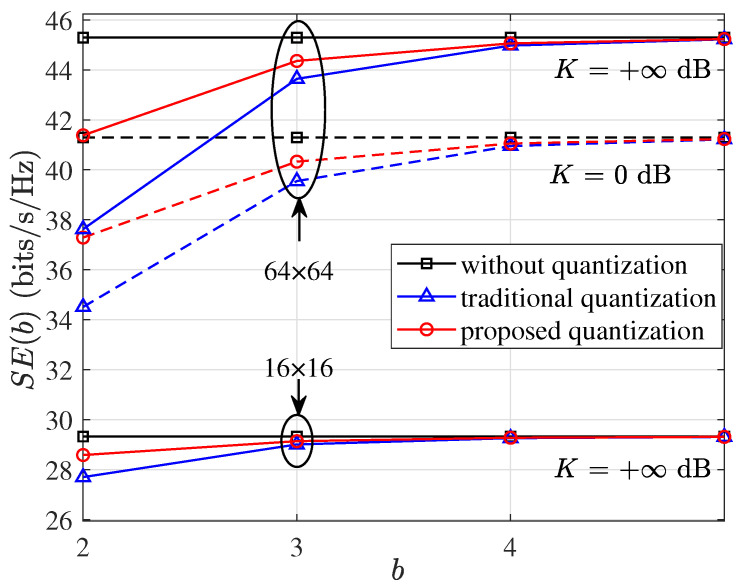
Spectral efficiency SE(b) versus quantization bits *b* with $\bar{\gamma }=10$ dB and R=50 m, where 16×16 and 64×64 indicate that there are 2×2 subarrays at the transceiver, each with 2×2 and 4×4 antennas, respectively.

Figure 3 compares the spectral efficiency SE(b) among different quantization strategies of using the designed codebooks, without quantization and with the quantization scheme in [24], where 2×2 subarrays (each with 2×2 or 4×4 antennas) and LOS channels are considered. It is shown that SE(2)≈28.60 bits/s/Hz, SE(3)≈29.15 bits/s/Hz and SE(4)≈29.30 bits/s/Hz, which becomes increasingly closer to the spectral efficiency without quantization, 29.32 bits/s/Hz in (2), with the increasing quantization bits. Targeting a loss of spectral efficiency within 5%, two quantization bits are needed for each subarray with 2×2 antennas, whereas three quantization bits are required for each subarray with 4×4 antennas. This indicates that the quantization bits for each phase angle are required to increase by one bit when the dimension of arrays is doubled. The number of bits required to properly quantize the phase angles increases with the increase in array size, as larger arrays produce narrower beams and require finer steering. The results in Figure 3 show that the designed codebooks are superior to that in [24] in terms of spectral efficiency using the same number of quantization bits. The performance under different Ricean factors *K* is also provided for comparison.

## 5. Numerical and Simulation Results

In this section, we present the numerical and simulation results to evaluate the LOS MIMO system performance using hybrid arrays with planar subarrays at the transceiver. Unless stated otherwise, we considered hybrid arrays with $N_{ty}=N_{tx}=M_{ry}=M_{rx}=P=Q=2$ and the carrier frequency of 220 GHz. We chose $S_{tx}=S_{ty}=S_{rx}=S_{ry}=\sqrt{\frac{\lambda R}{2}}$ and $d=\frac{\lambda }{2}$ as the subarray separation for a practical array placement to achieve the optimal performance at $\beta =0^{\circ }$ and R=50 m. The average SNR after analog beamforming combining, $\bar{\gamma }$, was set to be 10 dB in the simulation.

**Figure 4:**
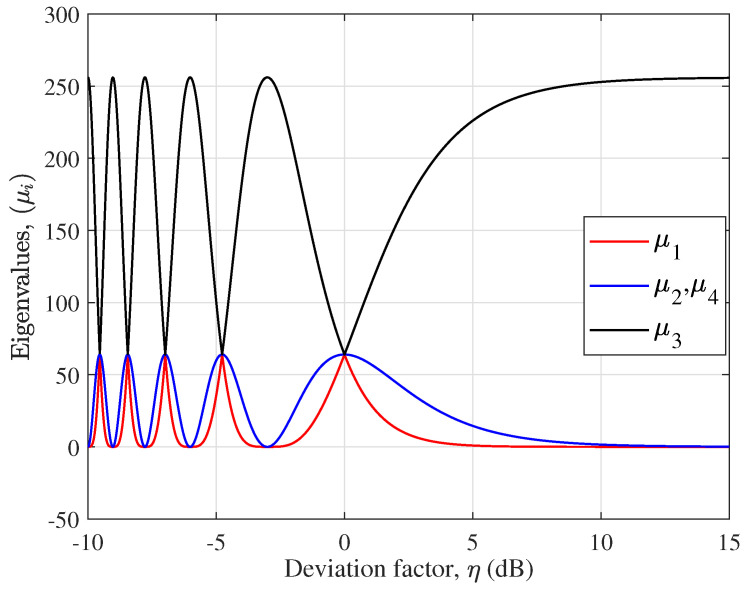
$\left\{\mu_{i}\right\}$ as a function of η in dB when $H=H_{LOS}$.

Figure 4 shows the eigenvalues $\{\mu_{i}{\}}_{i=1,\ldots ,4}$ versus the deviation factor η, where $\eta_{1}=\eta_{2}=\eta$ is assumed. There are only three eigenvalue curves in the figure due to $\mu_{2}=\mu_{4}$. It is found from (20) that the optimal performance is achieved when η=0 dB, which corresponds to the smallest subarray separation product in practical applications. Substituting η=0 dB into (23)–(26), we have $\{\mu_{i}{\}}_{i=1,\ldots ,4}=64$, which is consistent with the simulation results. When η>0 dB, the LOS MIMO channels will approach the statistical characteristics of SISO channels. The associated eigenvalue $\mu_{3}=16P^{2}Q^{2}=256$, and the others become zero when η→∞. When η<0 dB, the optimal performance can be achieved a few times corresponding to $\{\mu_{i}{\}}_{i=1,\ldots ,4}=64$ due to the periodicity of the trigonometric function in (17).

**Figure 5:**
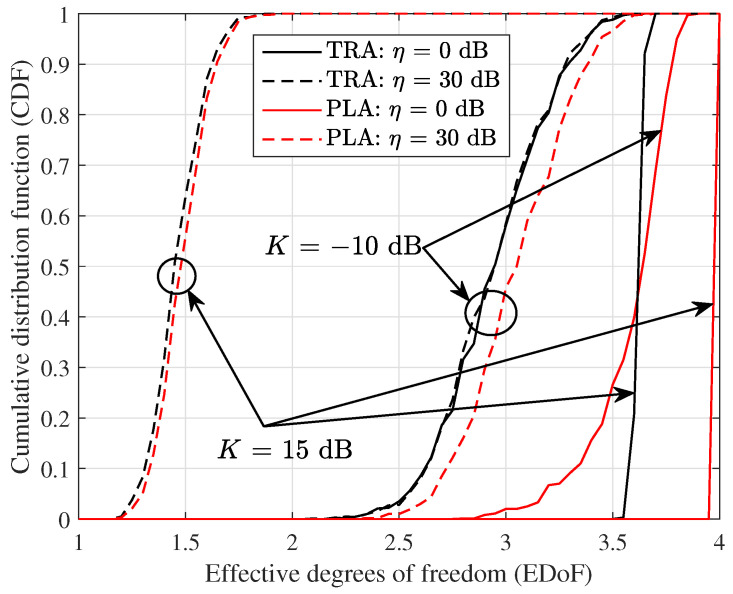
CDF of EDoF for different deviation factors η, and Ricean factors *K* with $\bar{\gamma }=10$ dB.

Figure 5 shows the cumulative distribution function (CDF) of EDoF for traditional array (TRA) and planar subarray (PLA) structures considering the optimal design in the analog domain and design mismatch, where traditional arrays are assumed to be the 2×2 antennas at the transceiver, respectively. It is observed that the two array structures have a similar trend of CDFs under the same conditions, but planar subarrays are always superior to traditional arrays in terms of EDoF. When K=−10 dB, i.e., the channel approaches Rayleigh fading, traditional arrays exhibit a close performance under different η, whereas there is an obvious difference for planar subarrays, which means that it is sensitive to η. When K=15 dB, i.e., the channel approaches a pure LOS condition, the EDoF becomes more sensitive to η. It is seen that LOS channels with an optimal subarray separation product are superior in term of EDoF compared to MIMO systems based on independent identically distributed Rayleigh channels.

**Figure 6:**
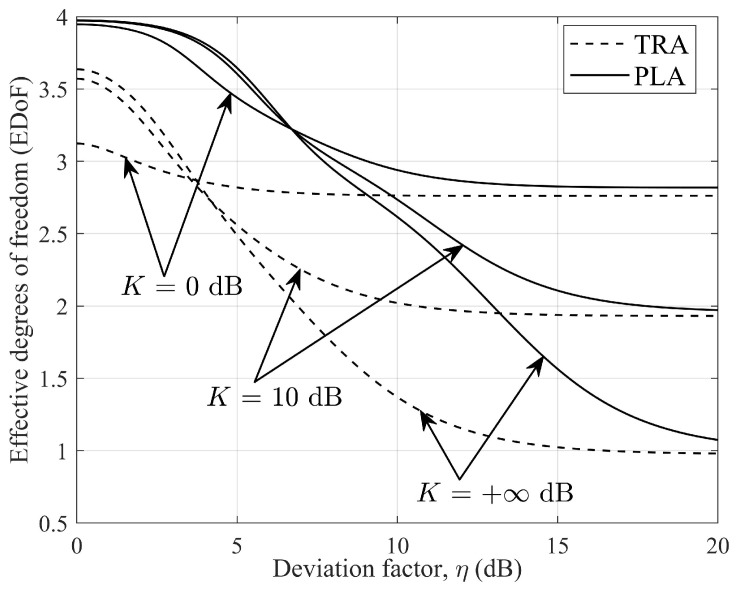
EDoF as a function of η, with $\bar{\gamma }=10$ dB and different Ricean factors *K*.

**Figure 7:**
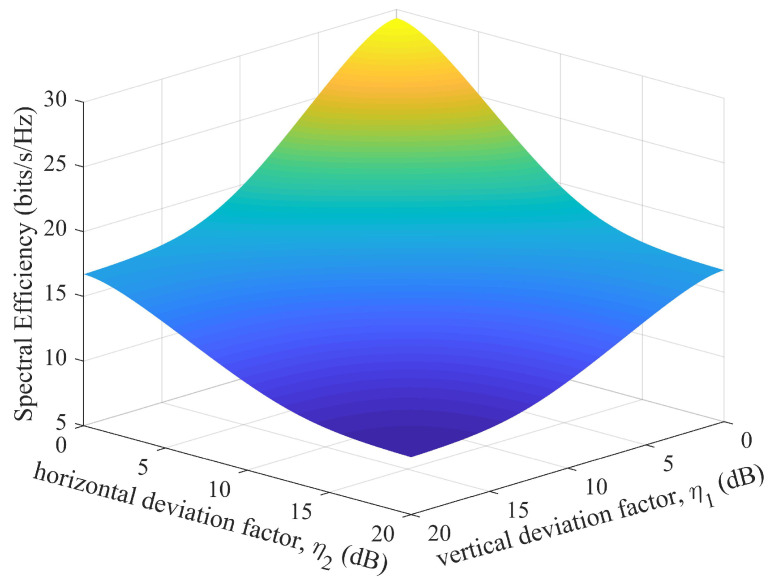
Spectral efficiency for different $\eta_{1}$ and $\eta_{2}$ with $\bar{\gamma }=10$ dB when $H=H_{LOS}$.

In Figure 6, the EDoFs are plotted as functions of η with different Ricean factors *K*. It is shown from this figure that the EDoF becomes increasingly dependent on η with the increase in *K*. It is also interesting to note that the EDoF using planar subarrays is almost independent of *K* when η≈7 dB, whereas η≈4 dB for traditional arrays. Under the same conditions, using planar subarrays can achieve a higher EDoF than traditional arrays. This figure reveals that, for the traditional array structure, the best performance for K=+∞ can be obtained only when the corresponding parameters meet the Rayleigh distance criterion. The EDoF will be degraded once η deviates from the optimal design in the analog domain. However, for the planar subarray structure, it becomes less sensitive when the parameters deviate from the optimal design in the analog domain within a certain range. This means that the planar subarray structure is more robust to the design mismatch. The minimum and maximum EDoFs in (27) and (28) were calculated as $EDoF_{\max}\approx 3.9752$ and $EDoF_{\min}\approx 0.9984$, respectively. For traditional arrays, $EDoF_{\max}\approx 3.6364$ and $EDoF_{\min}\approx 0.9756$. The above calculations are consistent with the simulation results in Figure 6. Figure 7 shows the 3D graphics of the system spectral efficiency as the function of $\eta_{1}$ and $\eta_{2}$ in a pure LOS MIMO channel.

**Figure 8:**
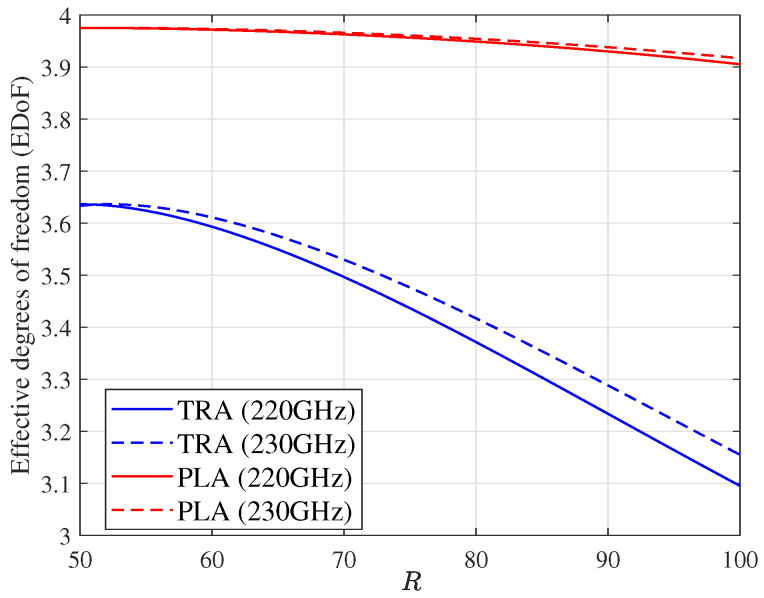
The comparison of EDoF for different *R*s and carrier frequencies with $\bar{\gamma }=10$ dB.

Figure 8 investigates how sensitive the EDoF is to the communication distance *R* for different carrier frequencies. It can be seen that the EDoF decreases with increasing *R*, but it falls more slowly using planar subarrays than traditional arrays. The EDoF of the former is always higher than that of the latter. These indicate that the use of planar subarrays not only reduces the sensitivity of EDoF to the change in *R*, but also achieves a higher EDoF under the same conditions compared to traditional arrays in LOS MIMO systems. We can also see that the system EDoF using planar subarrays is also less sensitive to the change in carrier frequency compared to using traditional arrays.

**Figure 9:**
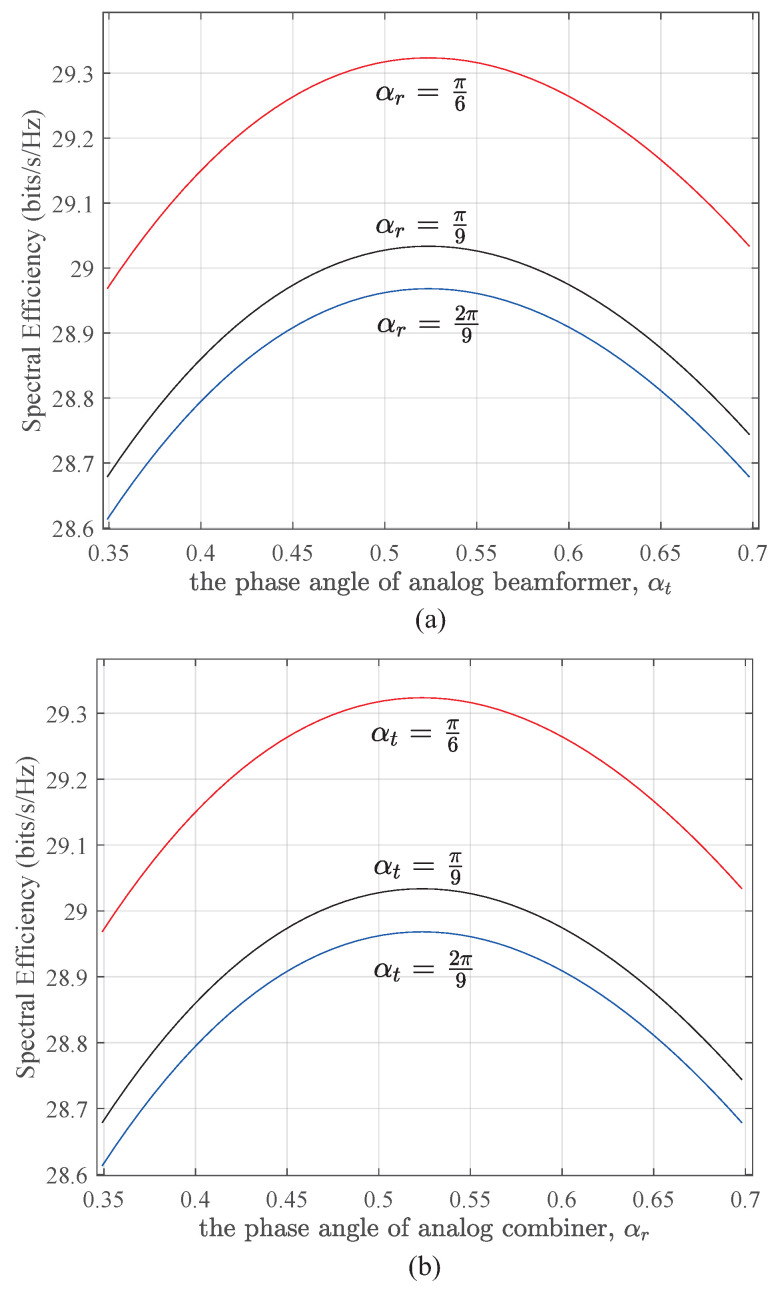
The relationship between spectral efficiency and phase angles of analog beamformer (**a**) and combiner (**b**), respectively.

Figure 9 shows the spectral efficiency as a function of phase angles $\alpha_{t}$ and $\alpha_{r}$ in the analog beamformer and combiner, respectively, where the phase angles $\phi_{t}$ and $\phi_{r}$ are assumed to be equal to π/3 for simplicity. We also assume the elevation and azimuth angles of the signals, $\theta_{nt}=\theta_{mr}=\pi /6$ and $\varphi_{nt}=\varphi_{mr}=\pi /3$, ∀n, *m*. Through a comprehensive analysis of the above two figures, it can be observed that the system spectral efficiency achieves the maximum value when $\alpha_{t}=\alpha_{r}=\pi /6$, which corresponds to the main beam directions of all subarrays directed at the directions of incoming signals. The large mismatch of phase angles will cause a serious loss in the system spectral efficiency.

## 6. Conclusions

Based on the introduced joint plane-wave and spherical-wave 3D channel model for hybrid arrays with planar subarrays, we investigated the spatial multiplexing performance of mmWave LOS MIMO systems and derived the optimal subarray separation products in the vertical and horizontal directions with respect to maximizing the spectral efficiency and EDoF. It is shown that the subarray separation products can be represented as a function of the communication distance, carrier wavelength, the number of subarrays and the angles of the array placement. Compared to traditional arrays, a planar subarray structure can not only achieve a higher EDoF but can also be more robust to the change in the communication distance and the NLOS components. Therefore, using the planar subarray structure can remove the limitation that the optimal performance is achieved only when the Rayleigh distance criterion is satisfied for traditional arrays. It is also found that the influence of the carrier frequency on the system EDoF in planar subarrays is lower than that in traditional arrays. With the designed quantization codebooks, it was found that, when the dimension of arrays is doubled, the number of quantization bits of each phase angle needs to be increased by one to maintain the performance. In future work, the considered system models can be extended to a multi-user case for more general applications by exploiting the user separation in the angular domain. How to further develop the joint optimal design approaches for analog–digital hybrid beamforming, e.g., exploiting the iterative method in [37] for multi-user LOS MIMO systems, will be an interesting future research topic.

## Data Availability

Not applicable.
